# Incubation in Various Diseases

**Published:** 1882-05

**Authors:** 


### Incubation in Various Diseases.

Dukes has carefully observed the duration of the stage of incubation in various infectious diseases. In 15 cases of scarlatina in which the length of this period could be determined, it was found to vary from 1 to 9 days, most cases occuping 2, 3, or 4 days. In 15 cases of varicella the duration was 13 to 19 days, the average being 15 or 16 days. In 42 cases of mumps the stage of incubation lasted on an average 19 days, but in some cases fell to 14, in others rose to 25 days. In 12 cases in which orchitis supervened, it occurred in most instances at an interval of 7 or 8 days after the inception of the mumps, although in one case it occurred on the second day. In 25 cases of Rötheln, the duration of incubation was from 12 to 22 days, most cases taking about 16 days to develop.

